# Protection against Fraudulent Malpractice Suits

**Published:** 1899-04

**Authors:** Bernard Wolff

**Affiliations:** Atlanta, Ga.


### PROTECTION AGAINST FRAUDULENT MALPRACTICE SUITS.*

By BERNARD WOLFF, M.D.,

Atlanta, Ga.

Physicians, as a class, are peculiarly exposed to attempts at blackmail, or the illegal and unjust extortion of money. The fact that the cure of disease does not, as a rule, reside in the doctor himself, but in his greater or less skill in directing the reparative powers of nature to the desired end, is not clearly appreciated by the great mass of the laity. The less informed among them endow the doctor with attributes little short of godlike, and consider him as holding the balance of life and death. If a patient succumbs to a malady not generally accounted fatal, or recovers from some violent traumatism with a shortened or stiffened limb, or with curtailment of bread-winning capacity, the medical attendant is apt to be subjected to sharp criticism. It needs but the Machiavellian touch of some Pariah of the law to convert this critical attitude into one of open opposition and accusation against the doctor. Legal proceedings are then threatened, to the end that grief, pain and disability may find assuagement in dollars and cents. The plaintiff avers that the bad termination of the case was due to the doctor's ignorance, neglect or error, and, conse-

*Read before the Atlanta Society of Medicine.

quently, the entire responsibility falls upon him, and he should be made to pay damages for his dereliction.

There may be instances here and there of malpractice suits being justified by facts, but they are few. It may be said, to the credit of the medical profession, that they who practice it, as a general rule, have a high regard for the responsibilities vested in them, and are conscientious in fulfilling their duties to the best of their knowledge and ability. For this reason damage suits are most often actuated by misconception of the doctor's attitude toward disease, or by malice and spite, or by fraud, and it is against the last that it behooves us to make some effort at the establishment of organized protection. Legal actions, brought with intent to defraud, are tantamount to blackmail, and blackmail is the lowest order of theft, for it combines the stealth and slyness of the sneak-thief with the bolder tactics of the burglar. Suits of this character are brought with no other motive than to obtain money under threat or by coercion. The wily shyster, whose aid is essential to the perpetration of the fraud, knows that the doctor's sole stock in trade is his reputation, and if that be placed under a cloud of suspicion his means of livelihood is seriously menaced. He knows, too, the defendant's aversion to legal processes, and that rather than undergo the expense of litigation and suffer the loss of patronage through publicity and scandal, he will prefer to pay a sum of money to have the suit quashed—"to compromise," in the euphemistic language of the courts. The physician is well aware that all will read in the newspapers, with infinite relish, the sensational charges against him, while few will notice his modest rebuttal, for this is the day when the newspaper has notoriously usurped the honorable function of the jury. So that, rather than sustain the injury to his good name and standing in the community, and to relieve himself of the annoyance and worry of constant threats, the doctor, in the end, generally tamely permits himself to be mulcted out of a sum of money by the rapacious shyster. A precedent is established in this way which will, in time, react on the submissive victim himself, as well as upon his medical fellows, and will serve to redouble the vigor of the legal quacks. It is a question whether these suits are ever intended to be brought to court. The civil docket is apt to be crowded and one, two or three years may elapse before the case is reached. But the same moral effect is obtained as if the suit were brought in good faith, and the shrewd and unscrupulous attorney uses this knowledge to his advantage and the great disadvantage of the defendant. Limp and characterless submission is demonstrably ill-advised. If the object of the fraudulent action takes a bold stand and bids defiance to his assailants, especially if he is protected in one of the ways presently to be mentioned, it is more than probable that high-handed proceedings and demands for colossal damages will shrink into cringing appeals for a pittance.

It is a fine confirmation of the rarity of gratitude that suits of this character are often brought by those who have been the beneficiaries of medical charity.

There are other schemes of blackmail which take the form of accusation against the doctor, of making improper proposals to a female patient or immoral assaults against her person when she is not in a position to defend herself, as when anesthetized. It is readily seen how easy it is to manufacture such accusations and how difficult they are to rebut.

A woman may enter the privacy of a doctor's office and suddenly make an outcry sufficient to attract attention, and if the victim does not at once stifle her outcry with money, she renews her clamor, and then tearfully and hysterically announces that the doctor has taken gross advantage of her ignorance or innocence, and has made attempts upon her chastity. If there be a confederate conveniently placed, who may be subsequently utilized as a witness, the difficulties and perplexities of the situation are greatly increased. This is what is called the badger-game, and is as dangerous as it is often successful.

Although the effect of anesthetics in arousing erotic sensation in women is well known, more than one doctor has been professionally injured, even imprisoned, on account of charges brought in mistaken good faith, or trumped up by women, of having taken extreme and unwarranted liberties with them when they were under the influence of a narcotic drug or an anesthetic. The presence of a third person should always be secured when anesthetizing a female patient, as a safeguard against disagreeable contingencies.

A physician in our city recently had an experience with a woman who falsely accused him of taking advantage of her in his office, and while it terminated successfully for him, at the same time it cost him a round sum of money to retain counsel, to say nothing of the mental unrest and neglect of practice during the progress of the affair.

Those of the medical profession who are engaged in surgical or obstetrical work are the ones most liable to be made the objects of malpractice suits, brought by an unscrupulous patient, or by his or her next friend, aided, or perhaps instigated, by an equally abandoned lawyer who is willing to undertake the conduct of the case on speculation.

This class of legal procedure is little better than blackmail, for while there may be, and perhaps occasionally is, some slight foundation for charges, the animus of the suit is so obviously to extort money that it is not relieved from the imputation of fraud.

An ununited fracture, the unrelieving of a fractured limb, a burn from a hot-water bottle or chemical cauterant, a lacerated perineum, and like frequent, and often, even in the most careful hand, unavoidable accidents, are made grounds for a suit for damages. Under the skilful modeling of an unscrupulous attorney the accident is made to assume stupendous proportions, and a story is woven from small incidents calculated to arouse the indignation of the community, that such a mass of stupid and brutal ignorance as this doctor should be allowed to remain undisturbed in its midst. In some parts of the country suits against surgeons are almost as common as against railroads and other corporations, and the result has been that the physicians there are disposed to abandon surgery or to entrench themselves behind written contracts before undertaking any operation. I venture to say that this condition of affairs is almost as bad in Atlanta. There are here very few physicians of standing who have not had experience with the blackmailing patient and his shyster counsel, and who do not retain unpleasant recollections of the worry and annoyance undergone.

There are several ways in which protection may be secured against these harpies.

One advantage that accrues from poverty, and poverty is all too familiar to the doctor, is the immunity it confers against speculative suits and blackmail. Most of us, however, would bravely resign ourselves to the risks which wealth entails if riches were offered us. Poverty is self-protective, but it is not desirable voluntarily to seek it, as its inconveniences somewhat preponderate over the protective feature. But even the poorest of us are sometimes assailed, fulfilling the saying of the Scriptures, "From him that hath not, it shall be taken away even that which he hath."

If the physician is fortunate enough to possess attachable property, it is perfectly right and legitimate for him to transfer it to those, the responsibility for whom and whose happiness and welfare are entrusted to his keeping. To conserve this obligation it is proper to convey to wife and children, or other near relative, all holdings subject to attachment; provided, of course, the motive of the conveyance be not fraudulent, or to invest his savings in United States government bonds, which are exempt. Security is thus bestowed and the title of the world's goods which he may have acquired by industry and economy, are not swept from him or from those dependent upon him.

Another safeguard would consist in having it illegal for an attorney to have an interest, direct or indirect, in a malpractice suit other than a fixed and unconditional fee. This would eliminate the speculative feature from these transactions and remove the chief source of fraudulent litigation.

It is an idle dream to hope to purge the courts of law of those fecal concretions in the form of shysters, which obstruct the current of justice, but some effort at disinfection should be attempted by attaching the penalty of disbarment to those who are parties to speculative and fraudulent suits against physicians.

An excellent plan to combat the schemes of unjust extortion is that suggested for medical men to contribute annually to the treasury of the State or local society a small sum to constitute a fund to be used in the defense of any member of the society who may be sued for malpractice; provided, after careful investigation, it is found that the defense is just. This fund is only available to those who are not pecuniarily able to engage counsel for defense. The knowledge of the existence of sinews of war would act as a strong deterrent to offenders, for they would recognize that their intended victim could not be frightened nor forced to give up money to save the large expense of employing counsel. It would be a great benefit to many who do not now, but may keenly feel the need of aid in time of trouble.

Poverty, conveyance of property, and combinations for mutual protection are our bulwarks of defense against perverted uses of the law.

As the first is desired of no man and at best negative, it is the least to be considered; but for the other methods, the time is ripe to carry them into effect, for without them who can be assured that he may practice his high calling without molestation, or can enjoy the fruit of his industry in peace?

